# Plants from Bulgarian Botanical Gardens: Some Selected Species with Potential for Health Food and Medical Applications

**DOI:** 10.3390/plants14142176

**Published:** 2025-07-14

**Authors:** Aleksandra Ivanova, Stefka Bogdanova, Veselin Petrov, Tsanko Gechev

**Affiliations:** 1 Center of Plant Systems Biology and Biotechnology, 4023 Plovdiv, Bulgaria; 2 Department of Plant Physiology, Biochemistry and Genetics, Agricultural University of Plovdiv, 4000 Plovdiv, Bulgaria; 3 Department of Molecular Biology, University of Plovdiv, 4000 Plovdiv, Bulgaria

**Keywords:** botanical gardens, medicinal plants, secondary metabolites, underutilized crops

## Abstract

Bulgarian botanical gardens harbor more than 3600 plant species from across the world. Some of them are well-known plants widely used by humans, others are underutilized crops or little-known exotic species. The latter group constitutes a rich reservoir of plant resources whose potential to bring benefits to society is still untapped. The aim of this review is to describe the diversity of species and their potentially valuable secondary metabolites in three of the largest Bulgarian botanical gardens, with a focus on underutilized crops and medicinal plants that are typical of Bulgaria. With this, we aim to pave the way for future research on the most promising of these plants. The report includes currently available ethnobotanical data on the properties and composition of their bioactive components, known culinary or therapeutic uses, and nutritional profiles. We also outline the vast potential of these plants in providing healthy diets, as well as for performing future groundbreaking biomedical research. Finally, we present the approach that will be used to screen extracts from these plants for biological activity.

## 1. Introduction

Plants are of paramount importance for food, feed, and materials for humans. In addition, they are known to be biochemical factories that produce more than one million metabolites [1]. Therefore, plants are a source of high-value metabolites with applications in medicine and cosmetics. More than 11% of the medications currently on the market have plant origins [2]. Yet, the vast potential of plant metabolites is still unexplored. The growing global population highlights the need for plant-based foods that support a healthy diet, along with innovative plant-derived medicines to address critical health conditions and disorders.

Botanical gardens harbor thousands of plant species across the world. While some of them are widely recognized and commonly utilized by people, many remain unstudied. In addition to their entertainment and educational value to society, botanical gardens play a vital role in biodiversity conservation by preserving genetic diversity and protecting endangered plant species. Although botanical gardens do not represent the full extent of plant life on Earth, they include approximately 30% of global plant diversity [3]. This makes them an ideal setting for exploring a broad spectrum of plants from diverse families, ecological niches, and geographic regions without the logistical challenges typically associated with field-based research.

Bulgarian botanical gardens have a vast concentration of species from all continents, cultivated under their optimal conditions (e.g., tropical rainforest environments, subtropical zones, arid environments, temperate climates in the northern and southern hemispheres). Some of these species are of great importance to ecosystems and/or mankind: major crops, edible plants, underutilized species, medicinal plants, and wild species with importance for ecosystems.

In this article, we first provide an overview of the species diversity in three of the largest botanical gardens in Bulgaria. Then, we focus on selected underutilized crops and medicinal plants that are typical of Bulgaria. These species have been selected on the basis of their traditional culinary use in Bulgaria and Europe, as well as on ethnobotanical data on their use in traditional and modern medicine. The goals of this study are to highlight their potential, to encourage their use as healthy food, and to stimulate further biomedical research. We discuss both their nutritional value and their metabolic diversity, particularly their unique secondary metabolites, which have medical implications. Moreover, we highlight the potential of some underutilized crops to supply essential minerals, vitamins, and fibers to diversify our diet. This is becoming increasingly important, as diversification of our food systems is directly linked to healthy living.

Finally, we propose a strategy to evaluate the biological activities of medicinal plants using model animal systems such as *Caenorhabditis elegans* [4]. Research using this model animal species is booming worldwide, with four Nobel Prizes in Physiology or Medicine having been awarded for scientific discoveries made working with *C. elegans*, most recently in 2024. In the section Future perspectives, we propose experiments that can utilize both the medicinal plants from these botanical gardens and *C. elegans* as a model system to study important human conditions and longevity.

## 2. Overview of the Plants in the Bulgarian Botanical Gardens

Here we review the plants in three Bulgarian botanical gardens managed by Sofia University: Sofia Botanical Garden, Varna Botanical Garden, and Balchik Botanical Garden (Table 1). The Sofia Botanical Garden has the largest number of plants, collected from across the globe and harbored in climate-controlled greenhouses with different ecosystems (tropical, arid, etc.). The Varna Botanical Garden has the largest area and harbors many tree species, whereas the Balchik Botanical Garden has both tree species as well as annual and perennial plants from across the globe.

**Table 1:** Bulgarian botanical gardens used in this study.

City	Year of Foundation	Area	Location	Altitude	Average Rainfall
Sofia	1892	0.5 ha	42°36′ N; 23°25′ E	549 m	650–700 mm
Varna	1977	36 ha	43°14′ N; 28°00′ E	28–85 m	450–550 mm
Balchik	1955	19 ha	43°24′ N; 28°01′ E	1–35 m	350–400 mm

This review focuses on the nutritional value and medicinal applications of key species native to Bulgaria. We conducted a comprehensive literature search using multiple scientific databases and platforms, including PubMed, Scopus, Web of Science, and Google Scholar. Both recent publications and older, foundational articles were considered to ensure a thorough and balanced overview of the topic.

**Figure 1:**
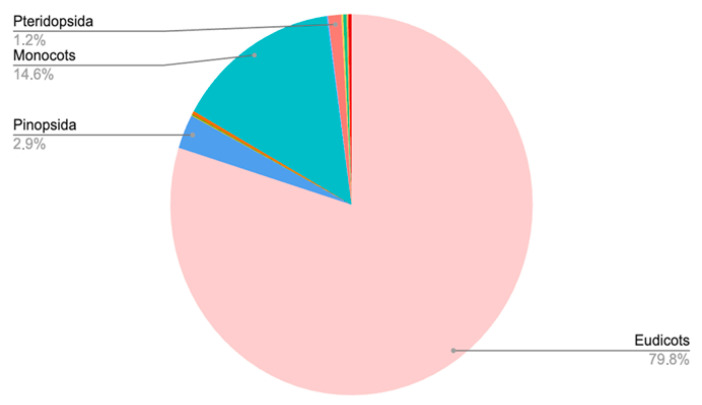
Taxonomic groups represented in the Bulgarian botanical gardens. From largest to smallest groups: Eudicots, Monocots, Pinopsida, Pteridopsida, mosses, and lycopods.

Overall, more than 3000 species are cultivated in the three botanical gardens, belonging to diverse taxonomic groups (Figure 1).

**Figure 2:**
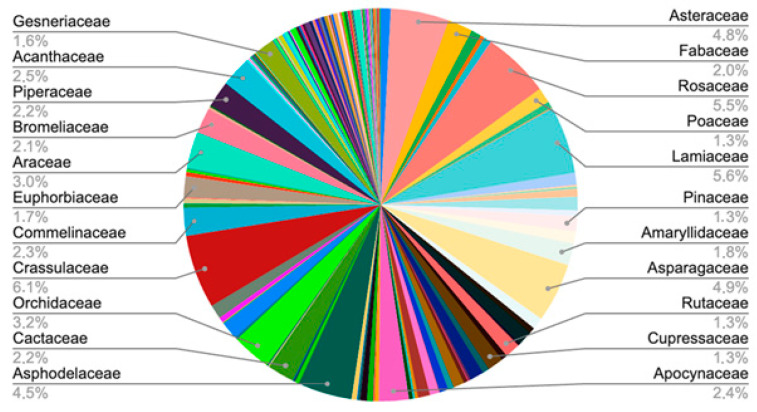
Distribution of distinct plant families in the Bulgarian botanical gardens.

Seed plants (Angiosperms, comprising Eudicots and Monocots, and Gymnosperms, mostly from Pinopsida) are by far the largest taxonomic groups, but other groups such as ferns (Pteridopsida), lycopods, and mosses are also represented (Figure 1). Among the seed plants, the most predominant group is the dicotyledonous species, with significant representation of the Crassulaceae, Lamiaceae, Rosaceae, Asparagaceae, Asteraceae, and Asphodelaceae families (Figure 2). All plant families and the number of plant species in them are given in Supplementary Table S1.

Whereas all three gardens have plant collections from all continents, the Sofia Botanical Garden has the highest number of medicinal plants and crop species from virtually all geographical areas and ecological habitats.

**Figure 3:**
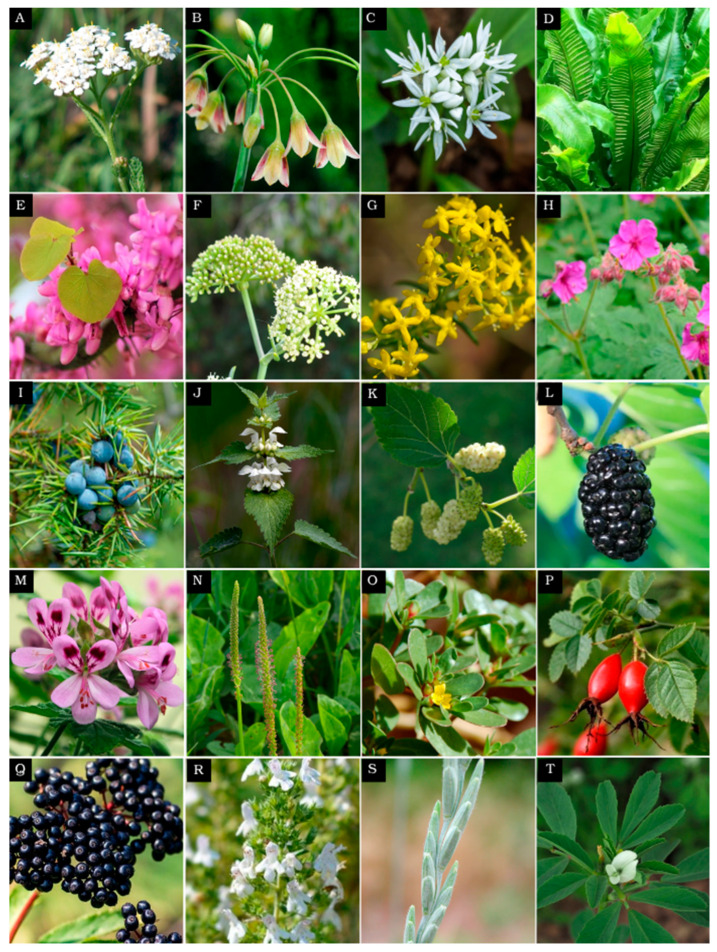
Bulgarian edible and medicinal species with their nutritional and medical benefits highlighted in this study. (**A**). *Achillea millefolium* L. (Common yarrow); (**B**) *Allium siculum* subsp. *dioscoridis* (Sm.) (**K**). Richt. (Bulgarian honey garlic); (**C**) *Allium ursinum* (Wild garlic); (**D**) *Asplenium nidus* L. (Bird’s nest fern); (**E**) *Cercis siliquastrum* L. (Judas tree); (**F**) *Crithmum maritimum* (Sea fennel); (**G**) *Galium verum* (Yellow bedstraw); (**H**) *Geranium macrorrhizum* L. (Bulgarian geranium); (**I**) *Juniperus communis* (Common juniper); (**J**) *Lamium album* (White dead-nettle); (**K**) *Morus alba* (White mulberry); (**L**) *Morus nigra* (Black mulberry); (**M**) *Pelargonium roseum* (Rose geranium); (**N**) *Plantago major* (Broadleaf plantain); (**O**) *Portulaca oleracea* (Purslane); (**P**) *Rosa canina* (Dog rose); (**Q**) *Sambucus nigra* L. (Elderberry); (**R**) *Satureja montana* (Winter savory); (**S**) *Thinopyrum intermedium* (Intermediate wheatgrass); (**T**) *Trigonella foenum-graecum* (Fenugreek).

Below, we provide an overview of a selection of important plants present in the botanical gardens, with a focus mainly on Bulgarian species (Table 2, Figure 3) and highlight their remarkable properties that spark scientific interest for further investigation. The underutilized crops presented here are chosen because they are already used in Bulgaria and a number of other countries. There are many more edible plants that can be further utilized as underutilized crops. Comprehensive studies of their nutritional properties (primary metabolites, essential elements, and vitamins) should be conducted in the future. The medicinal plants presented here are also a selection of Bulgarian species with ethnobotanical information about their medical use. We discuss their medicinal properties, and we share our ideas of how the model animal *C. elegans* can be utilized to study this potential for medical applications, e.g., alleviating obesity and improving longevity/healthy aging.

## 3. Underutilized Crops with Potential to Diversify Food Systems

Wild edible plants (WEPs), which can also be regarded as underutilized crops, represent an untapped reservoir of nutritional and bioactive compounds. These resilient, non-domesticated species have thrived in diverse environmental conditions, developing unique adaptations, including specific protective metabolites that may hold key health benefits [5]. Despite their historical significance in human diets, many WEPs have not been thoroughly investigated, leaving their full nutritional potential and bioactive properties unknown. Understanding WEPs and underutilized crops could not only expand our food sources but also contribute to the development of novel functional foods and sustainable agricultural practices, ensuring both safety and innovation in the culinary world. Here, we focus on selected representatives of neglected and underutilized crops present in Bulgaria (Table 2, Figure 3). These plants exhibit promising chemical compositions according to the existing literature, including essential amino acids, minerals, and vitamins, and further in-depth analyses could be beneficial to uncover their full potential.

***Allium siculum subsp. dioscoridis*** (*Sm.*) *K. Richt*. (Bulgarian honey garlic)

*Allium siculum* subsp. *dioscoridis* (Sm.) K. Richt., also known as *Nectaroscordum siculum bulgaricum* (*Allium bulgaricum*), is a traditional culinary spice from Southeast Europe, part of the Amaryllidaceae family [6,7]. Bulgarian samardala is a traditional spice made from the leaves of *N. siculum bulgaricum*, valued for its pungent, slightly bitter garlic–onion flavor [8]. Whether fresh in salads or dried as a salted blend, it is part of Bulgaria’s ethnobotanical heritage. Despite its unique features, it is poorly known in other countries and heavily underutilized.

Studies of *N. siculum bulgaricum* are very limited. A recent study on the chemical composition of samardala shows that it contains sulfur-containing metabolites such as dibutyl disulfide and S-methylcysteine sulfoxide, which are precursors of thiosulfinates—compounds known for their antimicrobial and anti-inflammatory properties [7,9,10]. It also highlighted its possible antioxidant potential, with total phenolic content ranging from 15.62 to 43.50 mg GAE/g DW and flavonoids ranging from 5.66 to 25.71 mg QE/g DW.

The plant also contains bioactive compounds such as hyperoside, kaempferol, sinapic acid, and p-coumaric acid, which are usually associated with anti-inflammatory, antioxidant, cardioprotective, and anticancer effects [7].

***Allium ursinum*** (Wild garlic)

*Allium ursinum* has been part of traditional Bulgarian cuisine since ancient times. Both the leaves and bulbs are used as salads, boiled as vegetables in dishes, pesto, soups, pasta, cheese, etc. [11,12]. It is known not only for its garlic-like scent but also for its ethnobotanical use in traditional folk medicine.

*A. ursinum* has been used since the Mesolithic era and was valued by ancient Greeks, Romans, and medieval herbalists for its medicinal properties [13]. The leaves and bulbs are traditionally used for digestive stimulation, antimicrobial effects, detoxification, and cardiovascular support [14,15]. It is also used for respiratory issues, and externally for wound healing, skin disorders, and acne [15].

In terms of its chemical composition, *A. ursinum* is rich in sulfur compounds, which are connected to its aromatic scent. Other compounds such as phenolics (gallic acid), flavonoids, steroidal saponins, as well as lectins, polysaccharides, and fatty acids are found [15,16].

Recent studies have investigated the antioxidant activity of *A. ursinum* in vitro, underscoring its potential [17]. These findings, combined with its long-standing role in traditional medicine, position *A. ursinum* as a versatile plant with a broad spectrum of possible applications.

***Crithmum maritimum* L.** (Sea fennel)

*Crithmum maritimum*, known as sea fennel, is an edible halophyte found in coastal habitats. It is gaining recognition as an emerging crop for biosaline agriculture because of its tolerance of high salinity, low nutrient availability, and other abiotic stresses [18]. As the demand for more sustainable and eco-friendly agricultural practices grows, sea fennel’s potential in the agri-food sector becomes increasingly significant. *C. maritimum* has been used in culinary dishes since ancient times because of its refined taste and distinctive aroma [19]. Its numerous positive biological properties have made it a staple in folk medicine, while its essential oil finds applications in cosmetology [19]. The GC-MS analysis of essential oils from *C. maritimum* identified sabinene (from 42.55 to 51.47%) and limonene (from 36.28 to 43.58%), as well as chlorogenic acid and its isomers (cryptochlorogenic and neochlorogenic acid) and other phenolics [20].

Sea fennel is a nutrient-dense plant, with its leaves, flowers, and fruits containing high levels of carbohydrates. In addition, its leaves are rich in omega-3 and omega-6 fatty acids, particularly linoleic acid. It is also rich in essential minerals such as sodium (Na), calcium (Ca), potassium (K), and phosphorus (P), contributing to its overall nutritional value. This plant’s extracts contain abundant antioxidants and polyphenolic compounds, which exhibit strong antimicrobial and antifungal activities against pathogens such as *Staphylococcus aureus* and *Candida* species. Additionally, sea fennel shows prebiotic effects, promoting the growth of beneficial gut bacteria such as *Lactobacillus bulgaricus*, while remaining non-toxic to human intestinal cells [21].

*C. maritimum* shows promising medicinal potential for liver diseases and metabolic health. Extracts inhibit liver cancer cell growth (HCC) and promote liver cell differentiation by reducing lactic acid fermentation. They also modulate key metabolic pathways, activating AMP-activated protein kinase (AMPK) and sirtuins (SIRT1 and SIRT3), thus improving cellular metabolism. *C. maritimum* extracts also prevent lipid accumulation in liver cells, offering a prospective approach for managing metabolic disorders [22]. The plant’s anti-inflammatory and antioxidant properties further enhance its therapeutic potential, supporting its use as a natural remedy for a variety of health conditions [23].

*C. maritimum* has also been investigated for its potential in agricultural pest control. Seed essential oil-based nanoemulsions effectively reduced *Spodoptera litura* (cotton leafworm) infestations by decreasing adult longevity and fecundity [24]. Additionally, it has promising features as a natural colorant [25].

These examples demonstrate that *C. maritimum* is an underutilized halophyte with many possible applications.

***Lamium album*** (White dead-nettle)

*Lamium album,* commonly known as white dead-nettle, is a flowering plant from the

Lamiaceae family [26]. In Bulgaria, *L. album* is also a part of traditional cuisine, with recipes passed down through generations. The stems and leaves are primarily used and can be consumed fresh, though they are more commonly prepared by briefly boiling the plant material (‘zaparka’) for 1–2 min before consumption or in soups. Bread from wheat with added *L. album* powder is rich in antioxidants [27]. Historically, *L. album* has been used as a food source during times of famine in Europe, China, and Japan, yet it remains underutilized in contemporary diets [28].

*L. album* contains a diverse array of bioactive compounds contributing to its therapeutic potential, recognized in traditional medicine. White dead-nettle has been used as an antiseptic, anti-inflammatory agent, and for its astringent properties, particularly in the treatment of menorrhagia, uterine hemorrhage, vaginal and cervical inflammation, and leucorrhea. For this reason, it is often referred to as the medicine for women’s ailments. Additionally, it has proven effective in managing chronic bronchitis and pharyngitis because of its mucolytic and antispasmodic activities [29,30]. Its chemical composition includes essential oils, phytoecdysteroids, terpenes, flavonoids (e.g., quercetin and rutin), iridoids, and phenolic acids [26,31]. Particularly, isoscutellarein derivatives and ecdysteroids found in the aerial parts are associated with anti-inflammatory, antioxidant, antibacterial, antiviral, and wound healing activities [32,33,34,35].

Given its rich phytochemical profile and broad therapeutic potential, *L. album* represents a promising yet underutilized nutritional resource, with potential applications in the development of dietary supplements and pharmaceutical agents.

***Morus alba*** (White mulberry)

*Morus alba* is one of the most abundant underutilized trees in Bulgaria. The fruits are eaten raw or used for jams, while the root bark and leaves have antimicrobial, antiviral, and antioxidant properties [36,37,38,39].

*M. alba* has the highest pH and soluble solids content among *Morus* species, indicating a sweeter taste and making it the most suitable for processing [40]. The fruit and leaves of white mulberry are rich in sugars, minerals, and bioactive compounds such as flavonoids and alkaloids, and have been developed as functional foods [41].

Different parts of *M. alba* have been used in traditional medicine. The leaves act as sweat inducers, cooling agents, and antipyretics, while the roots provide sedation, protection for the liver and kidneys. The fruits have diverse applications, including analgesic, antibacterial, and antihypertensive [42]. Additionally, *M. alba* is recognized for its benefits in cardiovascular diseases and its anticancer, anti-inflammatory, and neuroprotective properties [43]. New studies explore its appetite-suppressing and anti-obesity potential. Extracts from its root bark, enriched with Kuwanon G and Albanin G, showed strong CB1 receptor inhibitory activity, significantly reducing food intake, body weight, and metabolic disturbances in animal models. This suggests *M. alba* may be a promising natural agent for obesity treatment and metabolic syndrome management [44].

Further investigation could significantly deepen our understanding of the plant’s biochemical properties, mechanisms of action, and potential therapeutic applications. Expanding research efforts may not only validate its traditional uses but also uncover novel compounds and bioactivities, paving the way for new applications in medicine, nutrition, and sustainable agriculture.

***Morus nigra*** (Black mulberry)

*Morus nigra* L. (Black mulberry) is a valuable species within the *Morus* genus of the family Moraceae, traditionally cultivated for both ornamental purposes and its edible fruits [45]. Despite its long-standing cultivation for silkworm feed (*Bombyx mori* L.), *M. nigra* remains largely underutilized in agricultural production, particularly outside of several Asian regions where mulberry cultivation is more established [46,47]. Its fruits are commonly consumed fresh or processed into various food products, including jams, juices, syrups, dried fruits, and natural flavorings, particularly in Bulgaria and some other countries [45]. The species holds a notable place in traditional diets because of its nutritional value and medicinal applications.

Black mulberry is increasingly recognized for its nutritional value and rich phytochemical profile, contributing to its growing popularity in both fresh and processed food products. The fruit is particularly noted for its high content of bioactive compounds, such as phenolic compounds including flavonols and phenolic acids, as well as anthocyanins [45,47]. Among these, anthocyanins serve a dual role, imparting the characteristic dark pigmentation to the berries and contributing to their health-promoting properties [45]. These secondary metabolites exhibit antioxidant, anti-inflammatory, and potential antidiabetic activities, which provide the plant with ethnobotanical uses in Bulgaria as a treatment for anemia, hypertension, high cholesterol, constipation, immune support, and common colds [48,49]. Recent studies suggest that *M. nigra* may provide significant health benefits, particularly for individuals with type 2 diabetes mellitus, because of its phenolic composition. Similar beneficial effects have also been observed in mulberry leaves [50]. Another study has also explored the anticancer potential of *M. nigra* [51].

Due to its rich nutritional profile, diverse therapeutic potential, and established role in both traditional medicine and cuisine, *Morus nigra* remains a valuable yet underutilized species with significant promise for broader dietary and medicinal applications.

***Pelargonium roseum*** (Rose geranium)

In recent years, *Pelargonium roseum* has gained attention for its medicinal, sedative-hypnotic, therapeutic, and cosmetic properties [52].

The leaves of *P. roseum* are traditionally used as a culinary spice. The essential oil of *Pelargonium roseum* is known for its calming and relaxing effects, making it useful in aromatherapy to reduce stress and anxiety. In cosmetics, it helps moisturize and regenerate skin and tissues. Recent studies have also revealed its antimicrobial properties, although further research is needed since current findings are limited to in vitro experiments and animal studies [52,53].

Furthermore, due to the essential oil composition, this plant is also used for formulations against pests and parasites [54,55].

Thus, *P. roseum* is a versatile plant with numerous potential applications [56].

***Portulaca oleracea*** (Purslane)

*Portulaca oleracea* L. is a succulent annual plant with a cosmopolitan distribution, part of the Portulacaceae family [57]. It is also known as purslane, in Bulgarian “тученица” (tuchenitsa), and it can be found almost everywhere, often considered a weed.

*P. oleracea* is rich in nutrients and biologically active compounds and can be consumed both raw and processed. Recently, lactic fermentation has emerged as a promising biotechnological approach to enhance the content of bioactive compounds in *P. oleracea* puree [58]. *P. oleracea* is rich in valuable nutrients and bioactive compounds, particularly omega-3 fatty acids (notably α-linolenic acid) and vitamin C. Its leaves contain higher levels of α-tocopherol (vitamin E), glucose, fructose, and phenolic compounds such as oleracein A and C. Oxalic and organic acids are also more abundant in the leaves [59,60].

This chemical composition validates its traditional uses for digestive, respiratory, liver, and inflammatory issues, and its pharmacological effects such as antioxidant, analgesic, anti-inflammatory, and neuroactive [57,61,62].

***Rosa canina*** (Dog rose, Rose Hip)

The red-colored fruits of this plant are extremely popular in Bulgaria, both as a functional food and as a source of medications. The fruit is used in Bulgarian cuisine for jam and tea preparation. Deeply rooted not only in Bulgarian folk medicine, this plant has also been used in other countries to support heart health, metabolism, urinary and respiratory functions, digestion, hormonal balance, and wound healing [63].

*R. canina* is a valuable source of phytonutrients, such as vitamin C, tocopherols, phenolics, carotenoids, sugars, organic acids, and essential fatty acids [64]. It exhibits antioxidant and anti-inflammatory properties [65].

A recent study investigated the molecular and biochemical basis of petal color and scent in *R. canina*, revealing significant differences in bioactive compounds between white and dark pink petals. The research highlighted the flowers’ rich content of flavonoids, anthocyanins, and essential oils with health benefits, underscoring their potential as valuable natural products. The results open new avenues for genetic and biotechnological studies to maximize the medicinal and cosmetic potential of *R. canina* petals [66].

***Sambucus nigra* L.** (Elderberry)

*S. nigra* is both an edible and medicinal plant. The fruits can be eaten cooked or fermented; fermented elderberries, for example, are added to bread [67]. The flowers and fruits are used for immune support, flu, cough, diuretic, laxative, anti-inflammatory, respiratory, and antidiabetic effects. *S. nigra* can have antiviral effects against SARS-CoV-2 and Influenza A Virus [68]. It can also promote the healing of wounds [69].

*S. nigra* essential oil contains a balanced mix of saturated, monounsaturated, and polyunsaturated fatty acids—mainly oleic, palmitic, and linolenic acids—and is rich in volatile compounds such as nonanal and rose oxides. It shows significant antioxidant activity (IC_50_ = 2.52 mg/mL) and moderate antimicrobial effects, especially against *Candida albicans*. These bioactive compounds interact with microbial proteins, supporting the oil’s potential as a therapeutic agent for oxidative stress and infections [70].

***Satureja montana*** (Winter savory)

*Satureja montana* (Winter savory), a member of the Lamiaceae family, is a perennial aromatic plant traditionally used both as a culinary spice and a medicinal herb [71]. Winter savory is a native plant of southern Europe and is one of the most commonly used spices in Bulgaria, often cultivated in home gardens and found growing abundantly in mountainous regions [72]. All aerial parts of the plant are utilized, most commonly by drying and grinding them into fine powder. It is a traditional spice widely used in various Bulgarian dishes. *S. montana* is used in traditional medicine for treating digestive and respiratory issues [73]. Furthermore, antimicrobial, antibacterial, and antioxidant properties have been reported [74,75,76].

The ethnobotanical applications of *S. montana* are supported by a diverse range of metabolites [77]. These include four essential amino acids—L-valine, L-leucine, L-isoleucine, and L-phenylalanine—as well as six non-essential amino acids. The plant also contains various organic acids, with malic acid being predominant, alongside sugar acids and alcohols. Notably, linoleic acid has also been identified. Gas chromatography–mass spectrometry (GC-MS) analysis of the polar fraction II revealed the presence of nine phenolic acids, with 2,3-dihydroxyphenylacetic acid and 3,4-dihydroxyphenylacetic acid being the most abundant. Phenolic acids represent the most prevalent group of plant secondary metabolites, playing key physiological roles in plants and offering a range of valuable biological activities for humans. These include antioxidant, anti-inflammatory, antimicrobial, anti-allergic, hepatoprotective, anticarcinogenic, and antithrombotic effects, thereby supporting the traditional medicinal use of *S. montana* [78].

Further research into the chemical composition of *S. montana* could deepen our understanding of this plant and pave the way for novel applications in the food industry as a dietary supplement or as a potential therapeutic agent.

***Thinopyrum intermedium*** (Intermediate wheatgrass)

*T. intermedium*, or intermediate wheatgrass (marketed as *Kernza^®^*), is valued for its rich chemical composition and impressive nutritional profile. It has been used in culinary, mainly in baking, and is an underutilized crop, gaining more recognition [79,80,81,82,83]. This is a resilient perennial grain crop that is high in protein, fiber, vitamins, and antioxidants, and at the same time lower in starch content and deficient in high-molecular-weight glutenins [84].

These advantages make *T. intermedium* a valuable species not only for the agricultural sector but also as a subject of growing scientific interest because of its nutritional, ecological, and biochemical potential.

***Trigonella foenum-graecum*** (Fenugreek)

*Trigonella foenum-graecum* has a long history as a traditional medicine and natural food additive [85]. A traditional Bulgarian spice is made of the *T. foenum-graecum* seeds, called ‘sminduh’.

*T. foenum-graecum* has been used for its various health benefits in traditional medicine, including treating arthritis, asthma, bronchitis, digestive issues, reproductive and hormonal disorders, and menstrual pain. It is also used to promote wound healing, soothe sore throats, and regulate blood sugar [86].

Modern research highlights fenugreek’s wide range of pharmacological effects, such as antiatherogenic, antidiabetic, antianorexic, antioxidant, anticarcinogenic, antihyperlipidemic, and anti-inflammatory activities [85].

The plant is rich in proteins, carbohydrates, lipids, vitamins, and minerals [87].

**Table 2:** Bulgarian edible and medicinal species with their nutritional and medical benefits highlighted in this study.

Species Latin and English Names	Traditional Use, Used Parts, Major Nutrients/Valuable Metabolites, and Potential Applications	References
*Achillea millefolium* L. Common yarrow	Flowers and leaves are used in herbal infusions and cooking. Rich in essential oils, terpenes, and alkaloids, it exhibits strong antimicrobial and antioxidant properties, promising natural preservative.	[33,88,89,90,91]
*Allium siculum subsp. dioscoridis (Sm.) K. Richt.* Bulgarian honey garlic	Culinary spice. Similar to garlic, it contains thiosulfinates. Leaves are used to prepare the Bulgarian dish “sarmi”.	[6,7,8,9,10]
*Allium ursinum* Wild garlic	Both leaves and bulbs are used as salads, boiled as vegetables in dishes, pesto, soups, pasta, cheese, etc.	[11,12,13,14,15,16,17]
*Asplenium nidus* L. Bird’s nest fern	Used in folk medicine, the leaves and aerial parts contain flavonoids, phenolic acids, and xanthones, showing antioxidant, antimicrobial, and anticancer potential for drug development.	[92,93,94]
*Cercis siliquastrum* L. Judas tree	Flowers in folk medicine are used against anemia, malaria, and stress. Contains aldehydes, terpenoids, and flavonoids (catechin and myricetin). Used in cosmetics, agriculture, and potentially in cancer therapy.	[95,96,97,98,99]
*Crithmum maritimum*, Sea fennel	Emerging crop for biosaline agriculture. Leaves are rich in omega-3 and omega-6 fatty acids. Rich in essential minerals.	[18,19,20,21,22,23,24,25]
*Galium verum* Yellow bedstraw	Aerial parts are used in traditional Bulgarian medicine as an analgesic, laxative, astringent, diuretic, and local hemostatic. It has antioxidant and anti-inflammatory properties.	[100,101,102,103]
*Geranium macrorrhizum* L. Bulgarian geranium	Leaves, flowers, and roots are used in folk medicine for insomnia, high blood pressure, ulcers, wounds, inflammation, and nervous tension, with antibacterial and antiviral effects.	[104,105,106,107,108]
*Juniperus communis* Common juniper	Fruits are edible and are used for treating diabetes, arthritis, and digestive issues, with diuretic, anti-inflammatory, and antimicrobial effects.	[109,110,111,112,113,114]
*Lamium album * White dead-nettle	Whole plants are used in Bulgarian cuisine; they can be eaten raw or cooked. Known for its antiviral, antibacterial, antioxidant, and antidiabetic properties.	[26,27,28,29,30,31,32,33,34,35]
*Morus alba * White mulberry	Fruits are eaten raw or used for jams, while root bark and leaves have antimicrobial, antiviral, and antioxidant properties.	[36,37,38,39,40,41,42,43,44]
*Morus nigra * Black mulberry	Fruits are eaten raw or used in teas and jams. Leaves are valued for their anti-inflammatory properties. Leaves, fruits, and roots exhibit antinociceptive, antimicrobial, antidiabetic, and anti-obesity activities.	[45,46,47,48,49,50,51]
*Pelargonium roseum* Rose geranium	Leaves are used as a culinary spice. Essential oils support respiratory, digestive, and hormonal health, liver detox, and wound healing, with antioxidant and antibacterial properties.	[52,53,54,55,56]
*Plantago major* Broadleaf plantain	Leaves are used traditionally for wound healing, respiratory, digestive, reproductive issues, pain, and infections, with anti-inflammatory, analgesic, and antioxidant effects.	[115,116,117,118,119]
*Portulaca oleracea* Purslane	Stem and leaves are eaten raw or in salads; traditionally used for digestive, respiratory, liver, and inflammatory issues. Shows antioxidant, analgesic, anti-inflammatory, and neuroactive effects.	[57,58,59,60,61,62]
*Rosa canina * Dog rose	Fruits used in Bulgarian cuisine for jams and teas, traditionally for heart, metabolic, urinary, respiratory, digestive, hormonal, and wound healing support. Exhibits antioxidant and anti-inflammatory properties.	[63,64,65,66]
*Sambucus nigra* L. Elderberry	Fruits eaten cooked; flowers and fruits used for immune support, flu, cough, diuretic, laxative, anti-inflammatory, respiratory, and antidiabetic effects.	[67,68,69,70]
*Satureja montana* Winter savory	One of the most commonly used spices in Bulgaria. Also, used in traditional medicine for treating digestive and respiratory issues.	[71,72,73,74,75,76,77,78]
*Thinopyrum intermedium* Intermediate wheatgrass	Resilient perennial grain that is high in protein, fiber, vitamins, and antioxidants. Used in food products and baking, improves soil health, and reduces tillage.	[79,80,81,82,83,84]
*Trigonella foenum-graecum * Fenugreek	Traditional Bulgarian spice made of seeds; also used in traditional medicine. Rich in proteins, carbohydrates, lipids, vitamins, and minerals.	[85,86,87]

## 4. Medicinal Plants and Their Secondary Metabolites with Medical Applications

**Figure 4:**
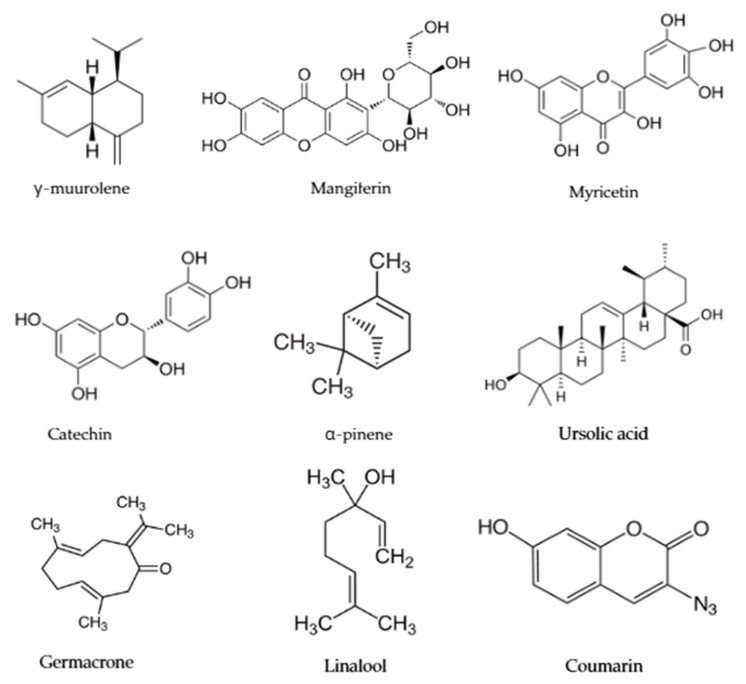
Metabolite compounds from selected plants with potential medical applications. γ-muurolene (*Achillea millefolium*); Mangiferin (*Asplenium nidus*); Myricetin (*Cercis siliquastrum*); Catechin (*Cercis siliquastrum*); α-pinene (*Juniperus communis*); Ursolic acid (*Plantago major*); Germacrone (*Geranium macrorrhizum*); Linalool (*Galium Verum*); Coumarin (*Galium Verum*).

The Bulgarian botanical gardens contain hundreds of medicinal species that have not been fully explored, with valuable secondary metabolites. Here, we review several of them, for which preliminary data indicate great potential for developing new medications (Figure 3), and we present some of their most prominent metabolites with medicinal properties (Figure 4).

***Achillea millefolium*** (Yarrow)

*Achillea millefolium*, commonly known as yarrow, is a highly adaptable plant from the Asteraceae family, often referred to as a “weed” because of its ability to grow in nearly any environment. Yarrow is easily recognized by its fern-like leaves and clusters of small white or pink flowers. It is used for its medicinal properties and distinctive taste and serves an important role in many Balkan recipes [89]. *A. millefolium* contains a variety of monoterpenes and sesquiterpenes, with monoterpenes being more abundant. The composition varies based on plant age and morphotype; for instance, camphene and limonene are only found in the white morphotypes, whereas α-pinene and β-myrcene are only found in the pink morphotypes. Other significant compounds include 1,8-cineole and γ-muurolene, which are common to all morphotypes. Specific morphotypes and collection points show variations in terpene contents. This variability in chemical content is influenced by ecological, climatic, and genetic factors, contributing to its diverse therapeutic properties [88].

*A. millefolium* has been part of traditional cuisine since ancient times. The flowers are used for making teas, while the leaves can be added to salads in small quantities. Yarrow is also used in vinaigrettes, marinades for grilled meat, and as an addition to dishes. In Germany and the Nordic countries, fresh yarrow leaves have historically been used as a hop substitute in beer brewing. Essential oils from yarrow, typically rich in terpinolene, 1,8-cineole, thujone, camphor, and borneol, exhibit antibacterial activity against pathogens including *Bacillus cereus*, *Enterococcus faecalis*, and *Serratia rubidaea* [89]. These results may suggest a new possible application of *A. millifolium* as a natural food preservative, offering an alternative to chemical preservatives in the food industry [90]. Bioactive compounds such as alkaloids, including azulene found in *A. millefolium,* offer anti-inflammatory and analgesic effects. A novel alkamide isolated from *A. millefolium* “Moonshine” leaves and stems has shown antimicrobial activity against *Propionibacterium acnes*, the bacteria responsible for acne. This alkamide reduces inflammation, scavenges free radicals, and decreases tyrosinase activity, helping with post-acne pigmentation [33]. These compounds contribute to yarrow’s antimicrobial potential, positioning it as a promising candidate for further clinical exploration, particularly in pain relief, anti-inflammatory applications, and wound healing.

Overall, *A. millefolium* is a versatile plant with culinary and medicinal value, known for its anti-inflammatory, antimicrobial, and antioxidant properties [91]. In the food industry, it could be explored as a natural preservative, offering a sustainable alternative to synthetic stabilizers by inhibiting harmful bacteria and supporting the demand for clean-label, natural ingredients.

***Asplenium nidus* L.** (Bird’s nest fern)

*Asplenium nidus*, commonly known as the “bird’s nest fern,” part of the Aspleniaceae family, has a long history of use in traditional medicine. Recent scientific studies reveal significant therapeutic potential of its bioactive compounds.

Research on two *Asplenium* species, *Asplenium adianthum-nigrum* and *Asplenium ruta-muraria*, has identified a rich content of biologically active compounds, predominantly flavonoids, phenolic acids, and xanthones. The analysis of these plant extracts has revealed the presence of the following key metabolites: flavonoids (rutin, epigallocatechin, and epicatechin), phenolic acids (gallic acid, caffeic acid, and ferulic acid), xanthones (mangiferin and mangiferin glucoside). It has been reported in the same study that both species show high antioxidant, antimicrobial, and antibiofilm activities because of the presence of flavonoids and phenols [92].

One of the most significant findings regarding *A. nidus* and other related species is their potential role in combating cancer. Isolated flavonoids from *A. nidus* (gliricidin-7-*O*-hexoside and quercetin-7-*O*-rutinoside) exhibited high cytotoxic activity and inhibitory effect on human cancer cell lines (human hepatoma HepG2 and human carcinoma HeLa cells). In addition, extracts of A. nidus have been tested against various bacterial strains and have demonstrated significant antimicrobial activity against multidrug-resistant pathogens. The flavonoids present in *A. nidus* exhibit strong antioxidant activity, as demonstrated by DPPH and ABTS assays. They effectively neutralize free radicals, reducing the risk of oxidative stress-related diseases, including cancer and neurodegenerative disorders [93]. Other studies show that acetone extracts of *Asplenium dalhousiae* and *Aplenium polypodiodes* have shown cytotoxic effects in time and dose-dependent manners against MDA-MB-231 (triple-negative breast cancer), by changing cell morphologies and decreasing cell viability [94].

To sum up, *A. nidus* stands out as a plant with immense pharmacological potential. Its bioactive compounds exhibit significant antioxidant, antimicrobial, and antiproliferative properties, making it a promising candidate for the development of new drugs and natural therapeutic agents.

***Cercis siliquastrum* L.** (Judas tree)

*Cercis siliquastrum*, commonly known as the Judas tree, is a deciduous tree from the Fabaceae family [95]. This ornamental tree is highly regarded for its vibrant, deep pink flowers and heart-shaped leaves and is widely planted in gardens and urban spaces. The tree’s small fruits are commonly reported to be edible and can be consumed raw or roasted. Additionally, young leaves and shoots are also mentioned in traditional use, often added to salads. However, further studies are still needed to assess their nutritional value and potential toxicity.

In traditional medicine, *C. siliquastrum* has been used to treat a variety of ailments, including anemia, malaria, and stress [96]. Despite its long history of use, gaps in knowledge regarding the chemical composition and biological activities of *C. siliquastrum* still persist. Further research is needed to explore the tree’s metabolomic profile, providing insight into its nutritional value and the possible health risks and benefits.

*C. siliquastrum* contains a variety of bioactive compounds in all of its parts. Key volatile compounds include aldehydes such as 2-hexenal and 2-methyl-4-pentenal, which comprise significant portions of the total volatile content in the leaves and flowers. The plant also contains alcohols, esters, terpenoids, and flavonoids, such as catechin and myricetin 3-*O*-rhamnoside, known for their antioxidant, antimicrobial, and anti-inflammatory properties. These compounds contribute to the plant’s therapeutic potential, particularly in its bark, which also contains volatile monoterpenes such as isoborneol and safranal. In traditional Iranian medicine, extracts from the leaves are used to treat many psychotic conditions. This therapeutic use may be linked to the plant’s high content of myricitrin [97]. In comparison, the flowers have been used for digestion improvement in Syria and as an antiseptic in Turkey, likely because of their high aldehyde and terpenoid contents, which are known to have antimicrobial and antioxidant properties. Its chemical composition may also offer anticancer potential by inducing apoptosis and inhibiting cancer cell growth, suggesting its potential role in cancer therapy [96,97]. *C. siliquastrum* shows promise for industrial applications, particularly in agriculture and cosmetics. The plant’s secondary metabolites, such as flavonoids and phenolic acids, serve protective roles against environmental stressors, functioning as natural UV filters and providing antimicrobial protection. These properties suggest potential applications in cosmetics, where extracts from the genus *Cercis* could be used to formulate UV-protective skin care products. Additionally, the plant’s antimicrobial properties could find use in agricultural applications, where it may serve as a natural pesticide or preservative [98].

From an ecological perspective, *C. siliquastrum* contributes to urban greening, serving as a biomonitor and improving biodiversity [99].

In summary, *C. siliquastrum* is a plant of scientific interest because of its diverse chemical composition and potential medicinal and industrial uses. Its rich phytochemical profile, especially flavonoids and volatile compounds, shows promise for treating health conditions and offers ecological and industrial applications.

***Galium verum*** (Yellow bedstraw)

*Galium verum* is a well-known and cherished mountain plant in Bulgaria. Its aerial parts have long been used in traditional Bulgarian medicine for their analgesic, laxative, astringent, diuretic, and local hemostatic properties [100]. It has also been mentioned in folk medicine for the treatment of hysteria and epilepsy, as well as a vulnerary (wound healer) [101].

A study examined the volatile compounds in fresh, dried, and essential oil forms of *Galium verum*. The fresh flowers’ aroma is mainly made up of monoterpenes, sesquiterpenes, esters, and other compounds. In dried flowers, the primary components are aldehydes, monoterpenes, alcohols, sesquiterpenes, esters, etc. [102].

The ethnobotanical use of *G. verum* has been scientifically validated, with studies confirming its pharmacological effects on the gastrointestinal and urinary systems, as well as its central nervous system activity, antibacterial and antifungal properties, and antioxidant potential [101,103].

***Geranium macrorrhizum* L.** (Bulgarian geranium)

*Geranium macrorrhizum* L. is one of the most popular ornamental plants in Bulgaria, traditionally cultivated in nearly every household garden. It has been used in folk medicine just as extensively as it has been cultivated, reflecting both its therapeutic value and cultural significance [104]. The leaves, flowers, and roots are used in folk medicine for insomnia, high blood pressure, ulcers, wounds, inflammation, and nervous tension, with antibacterial and antiviral effects [105,106].

The pharmacological properties of *G. macrorrhizum* have been extensively evaluated, demonstrating strong antioxidant activity confirmed by radical scavenging assays and high levels of phenolic and flavonoid compounds. Methanol extracts exhibited significant hepatoprotective effects in vivo, while the leaf methanol extract showed potent antibacterial activity, particularly against *Staphylococcus aureus* [107]. In another study, the antimicrobial properties of *G. macrorrhizum* were further explored, revealing strong and selective antibacterial activity against *Bacillus subtilis*, with minimum inhibitory concentrations (MICs) ranging from 0.4 to 1.0 μg/mL. Germacrone was identified as one of the principal bioactive compounds contributing to this effect [108].

These findings, along with its known astringent and antimicrobial effects, support its traditional use in folk medicine, especially for wound healing [107].

***Juniperus communis*** (Common juniper)

*Juniperus communis* is found in many home and rural gardens. Different parts of the plant are used in folk medicine for their diuretic, antiseptic, digestive, and anti-inflammatory properties. The berries and aerial parts have been applied in treating urinary tract issues, bladder and kidney conditions, menstrual disorders, and inflammation. The fruit has also been used as a stimulant and disinfectant, and in treating rheumatism, migraine, dropsy, and piles. In comparison, the bark has been used for pulmonary issues [109,110,111].

All these ethnobotanical applications can be attributed to the plant’s rich chemical composition, which includes flavonoids, such as apigenin, rutin, luteolin, and quercetin-3-O-arabinosyl-glucoside, d-α-pinene, camphene, pectins, glycolic, malic, formic, and acetic acids, as well as cyclohexitol. These compounds support various pharmacological activities, including hepatoprotective, antioxidant, analgesic, and antibacterial effects [111,112,113]. Studies of the properties of *J. communis* essential oils (EO) remain limited. In a recent study, EO extracted from *J. communis* cone-berries grown in Bulgaria was dominated by α-pinene (approximately 51%), with notable amounts of myrcene, sabinene, and β-pinene (around 5–6%). This α-pinene-rich chemotype demonstrated moderate anti-tyrosinase activity and significant antioxidant potential against ABTS•^+^ radicals [114].

Thus, *J. communis* is a valuable species attracting scientific interest because of its potential applications.

***Plantago major*** (Broadleaf plantain)

*Plantago major* is known for its medicinal use in many countries since ancient times, including Bulgaria [115].

Rich in diverse bioactive compounds, including phenol groups, organic acids, flavonoids, and terpenoids, with oleanolic acid and ursolic acid being the primary terpenoids found throughout the plant. It also contains essential fatty acids and carotenes, while its seeds are abundant in ferulic acid. Notably, ursolic acid acts as a selective inhibitor of cyclooxygenase-2, which likely underlies the plant’s well-known anti-inflammatory effects [116].

Together, these compounds contribute to the broad pharmacological potential of *Plantago major.* Traditionally, its leaves have been used to support wound healing and treat respiratory, digestive, and reproductive issues, as well as pain and infections, demonstrating anti-inflammatory, analgesic, and antioxidant activities. Additionally, *P. major* has been reported to exhibit anti-ulcerogenic, hepatoprotective, immune-enhancing, antidiarrheal, antinociceptive effects, and antibacterial effects [116,117,118,119].

## 5. Future Perspectives

The rich biodiversity in the Bulgarian botanical gardens presents opportunities for at least two clear avenues of future research: 1) Comprehensive metabolome and essential elements analyses of edible plants with potential to become crops. This analysis could identify edible plants rich in sugars, essential amino acids, minerals, and vitamins, which can diversify our diet and contribute to a healthy lifestyle worldwide. 2) The identification of medicinal plants with important health-promoting properties and potential applications in medicine and cosmetics. The plants can be utilized to establish comprehensive metabolite databases, which can be further explored by the scientific community worldwide. The biological activities of plant extracts and their metabolites can be explored using model organisms such as *C. elegans*. As a system that effectively translates findings from in vitro to in vivo systems [120], *C. elegans* offers several advantages, including a high degree of genetic similarity to humans, a wide array of available mutants, and a short lifespan that allows the study of multiple generations within a single year. These features make it particularly useful for investigating complex traits such as obesity and longevity. Previous studies have demonstrated that the plant flavonoid icariin protects against heat and oxidative stress, reduces fat accumulation, and extends lifespan in *C. elegans* [4]. Additionally, this model has been widely employed in neurodegenerative disease research, underscoring its utility in natural compound discovery [121].

**Figure 5:**
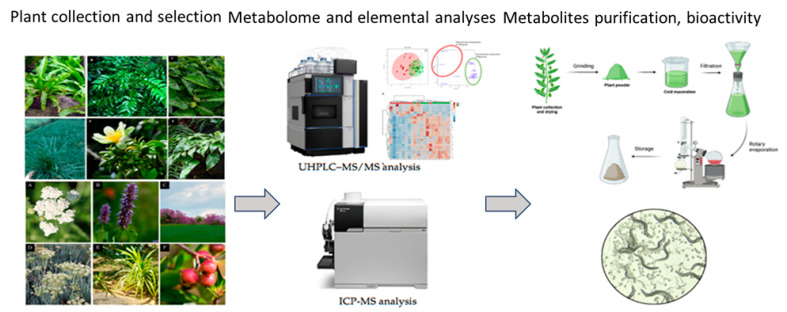
Future perspectives of the work on the plants from the botanical gardens.

Building on this foundation, our future work will focus on the bioactivity of lesser-known, underutilized, and ethnobotanically important plant species native to Bulgaria. Once species exhibiting beneficial effects, such as reduced fat storage or increased lifespan, are identified, their unique secondary metabolite profiles will be characterized using preparative HPLC or UHPLC-MS-based metabolomics and NMR spectroscopy (Figure 5). With this approach, hundreds of medicinal plants can be screened simultaneously with the aim of identifying new medications for healthy living. Finally, the evaluation of resilience and the key factors contributing to adaptability may provide clues for improving the tolerance of important crops, a crucial challenge faced by our community in an age of accelerating climate change. In this way, the rich biodiversity of Bulgarian botanical gardens can serve not only recreational and educational purposes but can also be a basis for future food science and biomedical research (Figure 5).

## 6. Conclusions

Bulgarian botanical gardens represent the rich global biodiversity of plants. They are a potential source of new healthy foods with an increase in nutrients, including minerals, vitamins, and other health-promoting compounds. Furthermore, some of these plants, especially those known from traditional medicine, can be sources of new metabolites with applications in medicine and cosmetics. In addition, the concentration of so many diverse species from different continents and biological habitats provides scientists with opportunities to conduct cutting-edge research without the need to travel to multiple global locations.

## Data Availability

The data presented in this study are available on the public website of the University Botanical Garden of Sofia University at https://www.ubg-bg.com/en/.

## Supplementary Materials

The following supporting information can be downloaded at: https://www.mdpi.com/article/10.3390/plants14142176/s1.
